# Association of gene polymorphisms with body weight changes in prediabetic patients

**DOI:** 10.1007/s11033-022-07254-y

**Published:** 2022-03-15

**Authors:** Farida V. Valeeva, Mariya S. Medvedeva, Kamilya B. Khasanova, Elena V. Valeeva, Tatyana A. Kiseleva, Emiliya S. Egorova, Craig Pickering, Ildus I. Ahmetov

**Affiliations:** 1 Department of Endocrinology, Kazan State Medical University, Kazan, Russia; 2 Laboratory of Molecular Genetics, Kazan State Medical University, Kazan, Russia; 3 Department of Biochemistry, Biotechnology and Pharmacology, Kazan Federal (Volga Region) University, Kazan, Russia; 4 Institute of Coaching and Performance, School of Sport and Wellbeing, University of Central Lancashire, Preston, UK; 5 Department of Physical Education, Plekhanov Russian University of Economics, Moscow, Russia; 6 Research Institute for Sport and Exercise Sciences, Liverpool John Moores University, Liverpool, UK

**Keywords:** Type 2 diabetes, Obesity, Diet, Single nucleotide polymorphism, Risk allele

## Abstract

**Background:**

Recent research has demonstrated that Type 2 Diabetes (T2D) risk is influenced by a number of common polymorphisms, including *MC4R* rs17782313, *PPARG* rs1801282, and *TCF7L2* rs7903146. Knowledge of the association between these single nucleotide polymorphisms (SNPs) and body weight changes in different forms of prediabetes treatment is still limited. The aim of this study was to investigate the association of polymorphisms within the *MC4R*, *PPARG,* and *TCF7L2* genes on the risk of carbohydrate metabolism disorders and body composition changes in overweight or obese patients with early carbohydrate metabolism disorders.

**Methods and results:**

From 327 patients, a subgroup of 81 prediabetic female patients (48.7 ± 14.8 years) of Eastern European descent participated in a 3-month study comprised of diet therapy or diet therapy accompanied with metformin treatment. Bioelectrical impedance analysis and genotyping of *MC4R* rs17782313, *PPARG* rs1801282, and *TCF7L2* rs7903146 polymorphisms were performed. The *MC4R* CC and *TCF7L2* TT genotypes were associated with increased risk of T2D (OR = 1.46, p = 0.05 and OR = 2.47, p = 0.006, respectively). *PPARG* CC homozygotes experienced increased weight loss; however, no additional improvements were experienced with the addition of metformin. *MC4R* TT homozygotes who took metformin alongside dietary intervention experienced increased weight loss and reductions in fat mass (p < 0.05).

**Conclusions:**

We have shown that the obesity-protective alleles (*MC4R* T and *PPARG* C) were positively associated with weight loss efficiency. Furthermore, we confirmed the previous association of the *MC4R* C and *TCF7L2* T alleles with T2D risk.

**Supplementary Information:**

The online version contains supplementary material available at 10.1007/s11033-022-07254-y.

## Introduction

Type 2 Diabetes (T2D) develops as a result of a complex interaction between adverse environmental and certain genetic factors [1]. At present, over 700 DNA polymorphisms have been identified that are associated with altered risk of T2D [2–4]; as such, T2D disease development is polygenic in nature [5].

It is well established that T2D may develop through various different pathways, including insulin resistance (IR) and beta-cell function deficiency, suggesting that different gene polymorphisms may be involved in T2D pathogenesis. These genes include *CDKAL1*, *CDKN2A*, *CDKN2B*, *TCF7L2*, *KCNJ11*, *UCP2*, *WFS1*, and *ABCC8*, amongst many others [6]. Conversely, an increased T2D predisposition may be driven by severe insulin resistance, which itself can be modified through polymorphisms of the *FTO*, *IRS1*, *PPARG*, and *PPARGC1A* genes [6]*.* Most of the identified genes associated with T2D affect the insulin secretion [7]. In the Russian population, genes that influence insulin synthesis and secretion in β-cells of the pancreas also appear to be the main driver in the development of T2D [8].

Effective, timely treatment of early carbohydrate metabolism disorders in order to prevent the future development of T2D is one of the most significant practical problems facing modern diabetology. The excess of abdominal body fat is a relevant risk factor for the development of over-inflammation/oxidative stress, which worsens the prognosis for further development of carbohydrate metabolism disorders [9, 10]. Thus, alongside advocating glycemic control, recommendations from various endocrinological associations highlight the importance of a reduction in patient body weight by 5–10% from the initial presentation [11, 12]. Previously, patients were exclusively educated around the principles of a balanced diet, without prescribing concomitant drug therapy. Recent research suggests that drugs from the biguanide group, and, in particular, metformin, may be effective in the early treatment of T2D. Metformin has a hypoglycemic effect, can help reduce body weight, and also serve to normalize lipid profiles [13, 14]. However, not all patients effectively respond to biguanide drug therapy [15–17], with one of the key drivers of this inter-individual variation in treatment response being identified as polymorphisms in the genes regulating metabolism [18, 19].

The aim of the present study was to explore the association of polymorphisms within *MC4R*, *PPARG*, and *TCF7L2* with the risk of different carbohydrate metabolism disorders and changes in the body composition in overweight or obese patients with early carbohydrate metabolism disorders.

### Ethics statements

The study was approved by the Local Ethics Committee of Kazan State Medical University (No 10 of 18.12.2018) and was carried out in accordance with the Declaration of Helsinki as revised in 2000. All the subjects provided informed consent before participating in the study.

### Participants

The study consisted of two parts: a case–control study and an intervention study. The case–control study involved 327 overweight or obese adults with T2D development risks (having first-degree relative with diabetes, history of cardiovascular diseases, hypertension ≥ 140/90 mmHg or undertaking therapy for hypertension, HDL cholesterol level < 35 mg/dL (0.90 mmol/L) and/or a triglyceride level > 250 mg/dL (2.82 mmol/L) in anamnesis, women with polycystic ovary syndrome, physical inactivity [11] and those who did not take medications that influence carbohydrate and fat metabolism. All of the patients underwent an oral glucose tolerance test and, as a result, 95 newly diagnosed prediabetic patients, 134 with the verified diagnosis of T2D, and 98 obese with normal glucose metabolism were identified. The average age at the time of the survey was 55.8 ± 12.7 years. Anthropometric parameters of all subgroups are shown in Supplementary Table 1. The control group included non-obese and non-diabetic healthy controls (all Caucasians of Eastern European descent and citizens of Russia).

### Oral glucose tolerant test

At the beginning of the study all participants underwent an oral glucose tolerance test (OGTT) with a standard carbohydrate load of 75 g dry glucose diluted in 300–400 mL, as recommended by the American Diabetes Association (ADA) for diagnostics of T2D and prediabetes. The criteria for carbohydrate metabolism disorders were due to the Standards of Medical Care in Diabetes of ADA [11].

### Intervention

**Fig. 1:**
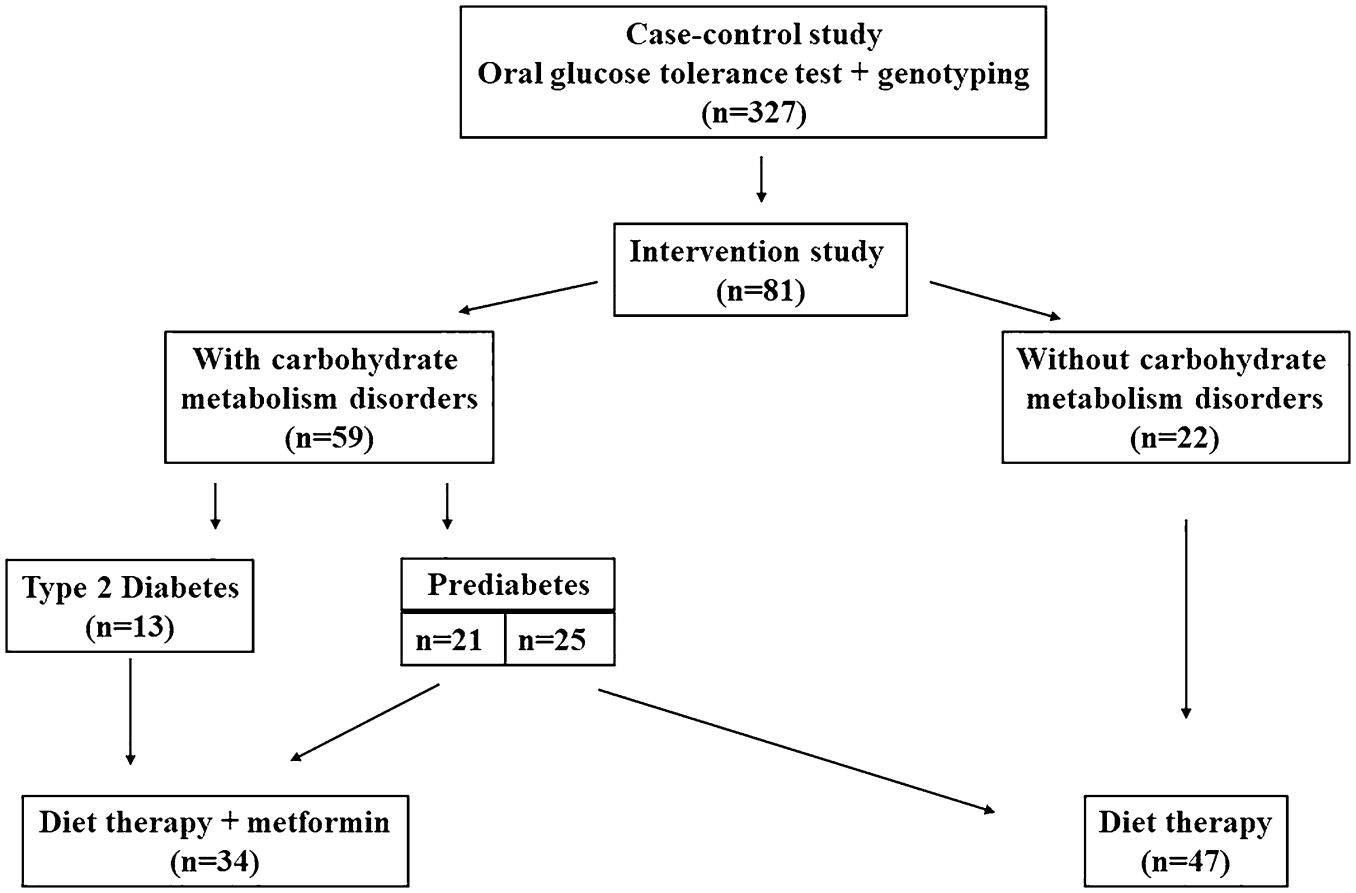
Study design

Out of the 327 patients, a subgroup of 81 female patients (48.7 ± 14.8 years) agreed to participate in a 3-month study comprised of further intervention (Fig. 1). Then the patients were divided into two groups utilising the simple randomization method. The first group comprised 47 patients (45.8 ± 14.8 years) undertaking diet therapy, which meant a balanced diet, with the exclusion of simple carbohydrate, and limiting complex carbohydrate and fat intake (the caloric intake was limited by 20 percent, and the macronutrient composition was comprised of 55% carbohydrate, 30% fat, and 15% protein). The second group comprised 34 patients (52.6 ± 14.2 years) who underwent the same diet therapy and 1500 mg/day metformin intake. The diet therapy guidelines and the dose of metformin were in accordance with Standards of Medical Care in Diabetes of ADA [11]. For the 3-month study period, once per week participant visits were performed, which involved food diaries checking.

### Bioimpedancemetry

At the beginning of the study and at 3-month follow up, all participants underwent bioelectrical impedance analysis with “DIAMANT-AIST” body composition analyzer (Saint-Petersburg, Russia). Changes in body mass, BMI, waist and hip circumferences, fat mass, total water and body cell mass were characterized.

### Genotyping

DNA was extracted from the blood samples of 327 participants using “AmpliPrime DNA-sorb-B” (NextBio, Moscow Russia), following the manufacturer’s recommended protocols. Nanodrop (ThermoFisher, USA) measurements were taken to measure the quality and quantity of the DNA. All samples were genotyped using allelic discrimination assays with TaqMan probes (Sintol, Moscow, Russia) on CFX96 Real-Time PCR Detection System (Bio-Rad, Hercules, California, USA). Assays were used for *MC4R* rs17782313 T/C, *PPARG* rs1801282 C/G, *TCF7L2* rs7903146 C/T, including appropriate primers and fluorescently labeled probes to detect the alleles.

An initial genotyping study of *MC4R* rs17782313 in 304 DNA samples, of *PPARG* rs1801282 in 324 DNA samples, of *TCF7L2* rs7903146 in 327 DNA samples was conducted. The distribution of genotypes and alleles of participants in the studied groups was compared with non-obese controls from the general (Russian) population: n = 172 for the *MC4R* rs17782313, n = 257 for the *PPARG* rs1801282, n = 404 for the *TCF7L2* rs7903146.

### Statistical analysis

Hardy–Weinberg equilibrium was tested by comparing the observed genotype frequencies with the expected frequencies using the Chi-square test with one degree of freedom in Microsoft Excel. Statistical analysis was conducted using GraphPad Instat. One-way analysis of variance (ANOVA) was applied to determine statistical significance among different groups. Paired t-tests were used to detect the significance of dynamic changes. Differences in phenotypes between groups were analyzed using unpaired t-tests. Body composition dynamics were calculated by percentage change of body composition parameters (% change from baseline) (Table 1). *p* < 0.05 was considered as statistically significant. Odds ratio with 95% confidence interval (CI) was used to assess the strength of the association of the investigated single nucleotide polymorphisms (SNPs)*.* Association analysis was also performed assuming co-dominant, dominant, and recessive models.

**Table 1:** Genotype and allelic frequencies of selected SNPs in patients and controls

Gene, rs	Group	n	Genotypes			*P*_1_	Risk allele, %	*P*_2_
*MC4R* rs17782313			TT	CT	CC		C	
	T2D	121	78 (64.5%)	33 (27.3%)	10 (8.3%)	0.05*	21.9	0.24
	Prediabetes	88	54 (61.4%)	29 (33.0%)	5 (5.7%)	0.46	22.2	0.32
	Overweight	95	54 (56.8%)	39 (41.1%)	2 (2.1%)	0.33	22.6	0.37
	Controls	172	92 (53.5%)	70 (40.7%)	10 (5.8%)	1.00	26.2	1.00
*PPARG* rs1801282			CC	CG	GG		C	
	T2D	131	93 (71.0%)	29 (22.1%)	9 (6.9%)	0.003*	82.1	0.07
	Prediabetes	94	69 (73.4%)	23 (24.5%)	2 (2.1%)	0.39	85.6	0.65
	Overweight	99	69 (69.7%)	25 (25.3%)	5 (5.1%)	0.03*	82.3	0.11
	Controls	257	192 (74.7%)	63 (24.5%)	2 (0.8%)	1.00	87.0	1.00
*TCF7L2* rs7903146			CC	CT	TT		T	
	T2D	131	73 (55.7%)	41 (31.3%)	17 (13.0%)	0.02*	28.6	0.04
	Prediabetes	91	44 (48.4%)	32 (35.2%)	15 (16.5%)	0.001*	34.1	0.001*
	Overweight	105	58 (55.2%)	32 (30.5%)	15 (14.3%)	0.01*	29.5	0.03*
	Controls	404	246 (60.9%)	135 (33.4%)	23 (5.7%)	1.00	22.4	1.00

*p < 0.05, statistically significant differences between participants with metabolic disorders and controls

## Results

### Case–control study

Genotype and allelic frequencies of three SNPs in patients and controls are shown in Table 1. The genotype distribution for each SNP was in agreement with the predicted Hardy–Weinberg equilibrium values (p > 0.05 in the T2D and control groups).

Genotype and allele frequencies of the *TCF7L*2 rs7903146 polymorphism differed significantly between type 2 diabetic patients and non-diabetic subjects (*p* = 0.02 and *p* = 0.04, respectively). The frequency of the risk (T) allele was 28.6% in T2D group and 22.4% in non-diabetic subjects, and this allele was significantly associated with T2D risk (OR = 1.39, 95% CI 1.01–1.90, *p* = 0.02). Moreover, the TT genotype was associated with a higher risk for T2D (OR = 2.47, 95% CI 1.28–4.78, *p* = 0.006). The T allele (OR = 1.66, 95% CI 1.05–2.63, *p* = 0.03) and TT genotype of the *TCF7L2* rs7903146 (OR = 3.27, 95% CI 1.63–6.55, *P* = 0.0005) were associated with prediabetes. The TT genotype of *TCF7L2* rs7903146 showed a significantly increased risk for obesity (OR = 2.76, 95% CI 1.38–5.50, *p* = 0.003). The risk genotype (CC) of *MC4R* rs17782313 SNP showed a significantly increased risk for T2D (OR = 1.46, 95% CI 0.59–3.62, *p* = 0.05). The C allele of *PPARG* rs1801282 SNP showed a significantly reduced risk for T2D (OR = 0.11, 95% CI 0.02–0.05, *p* = 0.0006) (Table 1).

### Associations between *MC4R, PPARG *and *TCF7L2* SNPs and weight loss efficiency

Participants in the diet therapy with metformin group showed a significant decrease in body weight (− 4.21 ± 0.67% vs. − 2.15 ± 0.48%; *p* = 0.01), BMI (− 1.77 ± 0.27% vs. − 0.87 ± 0.17%; *p* = 0.005), total water (− 0.38 ± 0.13% vs. + 0.02 ± 0.08%; *p* = 0.01) and body cell mass (− 0.44 ± 0.11% vs. − 0.15 ± 0.06%; *p* = 0.02) compared those participants in the dietary intervention only group (Table 2). There were no statistically significant differences in body composition changes between women with different menopausal status (Supplementary Table 2).

**Table 2:** Changes in body composition in 81 women (% change from baseline)

Parameter, %	Diet therapy	Diet therapy with metformin	*p*
Body weight	− 2.15 ± 0.48	− 4.21 ± 0.67	0.01*
BMI	− 0.87 ± 0.17	− 1.77 ± 0.27	0.005*
Waist circumference	− 4.25 ± 0.79	− 4.36 ± 0.86	0.90
Hip circumference	− 3.20 ± 0.87	− 4.10 ± 0.88	0.34
Waist/hip ratio	1.49 ± 0.72	− 0.51 ± 0.62	0.33
Fat mass	− 0.90 ± 0.20	− 1.20 ± 0.21	0.30
Total water	+ 0.02 ± 0.08	− 0.38 ± 0.13	0.01*
Body cell mass	− 0.15 ± 0.06	− 0.44 ± 0.11	0.02*

Data are Mean ± SEM

*p < 0.05, statistically significant changes after interventions

Genotype and allele frequencies of three SNPs in the intervention groups are shown in Supplementary Table 3.

Carriers of the *PPARG* rs1801282 CC genotype experienced a more substantial decrease in body weight (− 2.92 ± 0.57% vs. − 0.33 ± 0.70%; *p* = 0.013), waist/hip ratio (− 2.78 ± 0.97% vs. 0.70 ± 1.52%; *p* = 0.05) and BMI (− 3.51 ± 0.61% vs. − 0.22 ± 0.87%; *p* = 0.01) compared with G allele carriers in the diet therapy group. No differences in the diet therapy with metformin group were found (*p* > 0.05) (Table 3).

**Table 3:** The *PPARG* rs1801282 genotypes and body composition changes

Parameter, %	Diet therapy		*P*	Diet therapy with metformin		*P*
	CC (n = 33)	CG + GG (n = 14)		CC (n = 22)	CG + GG (n = 12)	
Body weight	− 2.92 ± 0.57	− 0.33 ± 0.70	0.013*	− 4.61 ± 0.83	− 3.53 ± 1.15	0.45
BMI	− 3.51 ± 0.61	− 0.22 ± 0.87	0.01*	− 1.92 ± 0.94	− 1.52 ± 1.39	0.49
Waist circumference	− 4.82 ± 1.02	− 2.49 ± 0.98	0.15	− 3.83 ± 1.14	− 5.25 ± 1.30	0.43
Hip circumference	− 3.10 ± 0.99	− 3.40 ± 1.8	0.86	− 3.79 ± 1.13	− 5.47 ± 1.41	0.37
Waist/hip ratio	− 2.78 ± 0.97	0.70 ± 1.52	0.05*	− 0.51 ± 0.82	− 0.53 ± 1.00	0.99
Fat mass	− 1.10 ± 0.25	− 0.36 ± 0.27	0.10	− 1.25 ± 0.25	− 1.15 ± 0.39	0.82
Total water	− 0.05 ± 0.10	+ 0.25 ± 0.16	0.09	− 0.48 ± 0.15	− 0.21 ± 0.23	0.34
Body cell mass	− 0.21 ± 0.07	+ 0.01 ± 0.08	0.10	− 0.54 ± 0.14	− 0.27 ± 0.20	0.29

Data are Mean ± SEM

*p < 0.05, statistically significant differences

Carriers of the *MC4R* rs17782313 TT genotype demonstrated a significantly greater reduction in body weight (− 5.35 ± 0.89% vs. − 2.5 ± 0.86%; *p* = 0.037), BMI (− 5.91 ± 0.95% vs. − 3.1 ± 1.22%; *p* = 0.044), hip circumference (− 5.98 ± 1.03% vs. − 2.07 ± 1.39%; *p* = 0.028) and fat mass (− 1.6 ± 0.28% vs. − 0.65 ± 0.26%; *p* = 0.027) compared with C allele carriers in the diet therapy with metformin group. No differences in diet therapy group were found (*p* > 0.05) (Table 4).

**Table 4:** The *MC4R* rs17782313 genotypes and dynamics of body composition changes

Parameter, %	Diet therapy		*P*	Diet therapy + metformin		*P*
	TT (n = 30)	TC + CC (n = 14)		TT (n = 21)	TC + CC (n = 14)	
Body weight	− 2.67 ± 0.59	− 1.03 ± 0.76	0.11	− 5.35 ± 0.89	− 2.5 ± 0.86	0.037*
BMI	− 3.19 ± 0.65	− 1.59 ± 0.87	0.16	− 5.91 ± 0.95	− 3.1 ± 1.22	0.04*
Waist circumference	− 4.56 ± 1.03	− 3.59 ± 1.16	0.57	− 5.48 ± 0.86	− 2.66 ± 1.66	0.11
Hip circumference	− 3.57 ± 1.09	− 2.41 ± 1.45	0.54	− 5.98 ± 1.03	− 2.07 ± 1.39	0.028*
Waist/hip ratio	− 1.28 ± 0.84	− 1.94 ± 1.40	0.67	− 0.20 ± 0.74	− 0.99 ± 1.11	0.54
Fat mass	− 1.09 ± 0.26	− 0.42 ± 0.26	0.12	− 1.6 ± 0.28	− 0.65 ± 0.26	0.027*
Total water	+ 0.006 ± 0.11	+ 0.06 ± 0.12	0.78	− 0.43 ± 0.19	− 0.3 ± 0.15	0.61
Body cell mass	− 0.17 ± 0.08	− 0.10 ± 0.09	0.62	− 1.03 ± 0.17	− 1.33 ± 0.15	0.60

Data are mean ± SEM

*p < 0.05, statistically significant differences

No association between the *TCF7L2* rs7903146 genotypes and body composition changes was found (Supplementary Table 4).

## Discussion

In this study we explored the effect of a dietary intervention comprised of the exclusion of simple carbohydrates and limiting of complex carbohydrates and fats on body composition in overweight or obese patients with early carbohydrate metabolism disorders, and the influence of polymorphisms in genes related with the risk of T2D on these outcomes. We confirmed the association of *MC4R* rs17782313 C and *TCF7L2* rs7903146 T alleles with the risk of T2D. Furthermore, we found that the protective alleles against obesity (*MC4R* rs17782313 T and *PPARG* rs1801282 C) were associated with weight loss effectiveness.

*TCF7L2* encodes a transcription factor in the Wnt signaling pathway. Nucleotide substitution (C to T) in this gene increases the risk of T2D [20]. The association of *TCF7L2* rs7903146 with the development of T2D is reproducible in many populations. In particular, in the present study of the Russian population, we revealed that the T allele of this polymorphism is more common in the participants with prediabetes, T2D and obesity. The same results were shown in a number of other studies, which demonstrate the association of *TCF7L2* rs7903146 with T2D in across many ethnic populations [21–23]. However, there appears to be no significant association between the T allele of *TCF7L2* rs7903146 and T2D risk in Chinese population [24].

The molecular mechanism of variation in *TCF7L2* and risk of T2D and prediabetes is currently unknown*.* However, some studies demonstrate involvement of *TCF7L2* in body weight regulation and the development of obesity and T2D due to impairment of β-cell function and then insulin secretion [25, 26]. The association of *TCF7L2* rs7903146 with the outcome of metformin treatment also remains poorly studied, and represents a subject for active discussion by researchers in the future. A systematic review of 34 studies of the Cochrane Library and EMBASE demonstrated that this polymorphism is not associated with the effectiveness of metformin treatment [27]. We also didn’t find any significant differences in body composition changes in studied groups due to the variants of *TCF7L2* rs7903146.

To date, most studies exploring the association of *MC4R* rs17782313 with risk of obesity and insulin resistance, high BMI and large waist circumference demonstrate an influence of the C allele [28–30]. It should be noted that melanocortin 4 receptor, which is encoded by *MC4R,* takes part in the regulation of insulin secretion [31], accordingly, nucleotide substitution (T to C) in the *MC4R* gene can lead to pathologies of lipid and carbohydrate metabolism.

This study demonstrates that the CC genotype of *MC4R* rs17782313 is more common in patients with T2D, but not prediabetes or obesity. Moreover, a number of studies have shown the association of polymorphisms within *MC4R* and the development of T2D [32]. Particularly, it was reported, that the *MC4R* rs17782313 C allele is most often associated with an increased T2D risk [33].

In our study, with adherence to diet therapy, C allele carriers and TT homozygotes of *MC4R* rs17782313 didn’t differ in body compositions changes, which is in agreement with the results of Diabetes Prevention Program (DPP) [34]. However, participants in our study who underwent metformin and diet therapy experienced a more substantial decrease in body weight and fat mass if there wereTT homozygotes. Although the effect of this polymorphism on the results of metformin therapy has not been previously studied, there is data demonstrating the association of *MC4R* rs9966412 and rs17066859 SNPs with loss of weight in patients while adding metformin to the treatment plan [35]. We suggest that it may be related with the action of melanocortin 4 on the production of insulin by the β-cells of the pancreas, as in case of monogenic form of obesity.

Among the known polymorphic markers of *PPARG*, rs1805192 is the most studied. The main role of the nuclear receptor superfamily PPARγ is the control of genetic expression, which influences the regulation of carbohydrate and lipid metabolism, adipogenesis, and synthesis of TNF-α, resistin and adiponectin.

At present, there is contradictory data on the association of *PPARG* rs1801282 with the development of metabolic syndrome (MS) and T2D. Accordingly, a number of studies demonstrated a decreased risk of MS and T2D in G allele carriers [36, 37]. In studies of other ethnic groups, this protective effect has not been found; indeed, the rs1801282 G allele was associated with increased risk of obesity in the populations of Spain, India and Mexico [38–41].

Cole SA et al. showed that the carriers of CG genotype are more predisposed to the increasing of BMI and development of obesity [40]. In contrast, Swarbrick et al., reported no association of CG genotype with obesity, arterial hypertension or T2D. Nevertheless, they made an interesting conclusion that lipid metabolism disorders are much more common among obese G allele carriers [42].

In our study, performed on a Russian population, we report results: the GG genotype was more common in patients with T2D, which is consistent with a number of studies on populations of Spain and India [38, 39]. However, the C allele was associated with a reduced risk of T2D. One potential explanation of this finding may be the relatively small number of GG genotype carriers.

We found that the C allele (protective against obesity) is associated with a more pronounced weight loss and decrease of waist/hip ratio (an indicator of abdominal fat tissue) among patients undertaking diet therapy, which is aligned with the results of earlier studies from Adamo et al. and Matsuo et al.; here, the G allele was more common among patients resistant to diet therapy [43, 44]. Furthermore, in DPP and the study of Frank et al., the *PPARG* rs1801282 G allele was associated with the short-term (up to 6 months) and long-term (up to 2 years) maintenance of achieved weight loss [34, 45]. We suggest that the resistance of G allele carriers to changes in body weight may be explained by the fact that the presence of this allele is associated with a decrease in the transcriptional activity of PPARγ [46, 47]. In this case, the *PPARG* rs1801282 C allele is associated with an increase in the transcriptional activity of PPARγ, which in turn leads to an increase in the sensitivity of cells to the action of insulin [48], promoting weight loss among patients. The absence of differences in weight loss between carriers of different alleles in the metformin plus diet therapy group may be explained by the suggestion that metformin may decrease the expression of *PPARG* [49].

The small number of subjects and the community setting, along with the exclusive use of female participants in the intervention study, all act as limitations to the present study. Also, it should be noted that other DNA polymorphisms and intervention methods such as exercise may potentially affect treatment outcomes [50]. In future, these limitations may be overcome by increasing of the number of participants, genetic markers, and including participants from both sexes.

## Conclusions

We confirmed the association of *MC4R* rs17782313 and *TCF7L2* rs7903146 SNPs with risk of T2D development. We also detected that *MC4R* rs17782313 and *PPARG* rs1801282 genotypes play an important role in body composition changes. CC homozygotes for *PPARG* rs1801282 appear to experience more pronounced weight loss; however, the addition of metformin to the treatment plan leveled any further changes in body weight. TT homozygotes for *MC4R* rs17782313 experienced increased weight loss and reductions in fat mass following the addition of metformin to diet therapy. According to our results, we suggest that these SNPs may be helpful in predicting the risk of T2D development and developing an optimal treatment plan in the early stages of the disorder. Further studies will show if the studied polymorphisms have a long-term effect on body composition changes.

## Supplementary Information

Below is the link to the electronic supplementary material.

Supplementary file1 (DOCX 32 kb)
