# Supplementary material for: Association of gene polymorphisms with body weight changes in prediabetic patients
Source: Mol Biol Rep. 2022 Mar 15;49(6):4217–24. doi: 10.1007/s11033-022-07254-y (PMC9262768; doi:10.1007/s11033-022-07254-y)
Supplement: Supplementary file 1 — Supplementary file1 (DOCX 32 kb) [file 11033_2022_7254_MOESM1_ESM.docx]

**ASSOCIATION OF GENE POLYMORPHISMS WITH BODY WEIGHT CHANGES IN PREDIABETIC PATIENTS**

**Supplementary Table 1. Anthropometric parameters of 327 participants**

| **Study group** | **Body weight (kg)** | **BMI (kg/m^2^)** | **Waist circumference (cm)** | **Hip circumference (cm)** | **Waist-to-hip Ratio** |
| --- | --- | --- | --- | --- | --- |
| T2D (n=134) | 86.2±0.1 | 32.2±0.03 | 103.6±0.09 | 110.7±0.08 | 0.94±0.0004 |
| Prediabetes (n=95) | 84.4±1.9 | 31.4±0.6 | 104.6±1.3 | 112.8±1.2 | 0.92±0.01 |
| Overweight (n=98) | 81.9±0.2 | 30.3±0.05 | 99.9±0.1 | 109.8±0.1 | 0.90±0.001 |

Data are Mean±SEM.

**Supplementary Table 2.** **Changes in body composition in therapeutic groups according to the menopausal status (% change from baseline).**

| **Parameter** | **Diet therapy** | | | **Diet therapy with metformin** | | |
| --- | --- | --- | --- | --- | --- | --- |
|  | **Menopause (n=21)** | **Fertile (n=25)** | ***P*** | **Menopause (n=23)** | **Fertile (n=12)** | ***P*** |
| Body weight | -1.94±0.49 | -2.31±0.78 | 0.71 | -4.39±0.79 | -3.85±1.29 | 0.71 |
| BMI | -2.72±0.71 | -2.64±0.77 | 0.93 | -4.98±0.94 | -4.44±1.41 | 0.75 |
| Waist circumference | -3.18±1.39 | -5.11±0.86 | 0.23 | -4.72±1.14 | -3.64±1.26 | 0.56 |
| Hip circumference | -3.38±1.43 | -3.05±1.09 | 0.85 | -5.45±1.19 | -2.42±1.02 | 0.11 |
| Waist/hip ratio | -0.23±1.10 | -2.50±0.92 | 0.12 | 0.11±0.76 | 1.28±1.09 | 0.38 |
| Fat mass | -0.72±0.21 | -1.00±0.32 | 0.50 | -1.17±0.24 | -1.30±0.42 | 0.77 |
| Total water | 0.03±0.13 | 0.01±0.11 | 0.91 | -0.45±0.15 | -0.24±0.24 | 0.45 |
| Body cell mass | -0.14±0.07 | -0.15±0.09 | 0.99 | -0.55±0.14 | -0.22±0.20 | 0.19 |

Data are Mean±SEM.

**Supplementary** **Table 3. Frequencies of genotypes and risk alleles of identified of 81 women.**

| **Groups** | ***PPARG* rs1801282** | | | |
| --- | --- | --- | --- | --- |
|  | **CC** | **CG** | **GG** | **C allele, %** |
| All of the participants | 67.9 | 25.9 | 6.2 | 80.9 |
| Diet therapy (n=47) | 70.2 | 23.4 | 6.4 | 81.9 |
| Diet therapy with metformin (n=35) | 64.7 | 29.4 | 5.9 | 79.4 |
|  | ***TCF7L2* rs7903146** | | | |
|  | CC | CT | TT | T allele, % |
| All of the participants | 41.5 | 47.5 | 11.0 | 34.8 |
| Diet therapy (n=47) | 40.4 | 53.2 | 6.4 | 33.0 |
| Diet therapy with metformin (n=35) | 42.9 | 40.0 | 17.1 | 37.1 |
|  | ***MC4R* rs17782313** | | | |
|  | TT | TC | CC | C allele, % |
| All of the participants | 64.6 | 30.4 | 5.1 | 20.3 |
| Diet therapy (n=47) | 68.2 | 25.0 | 6.8 | 19.3 |
| Diet therapy with metformin (n=35) | 60.0 | 37.1 | 2.9 | 21.4 |

**Supplementary** **Table 4. The *TCF7L2* rs7903146 genotypes and body composition changes.**

| **Parameter, %** | **Diet therapy** | | ***P*** | **Diet therapy + metformin** | | ***P*** |
| --- | --- | --- | --- | --- | --- | --- |
|  | CC (n=19) | CT+TT (n=25) |  | CC (n=15) | CT+TT (n=20) |  |
| Body weight | -1.97±0.67 | -2.27±0.66 | 0.80 | -3.98±0.97 | -4.38±1.31 | 0.77 |
| BMI | -0.75±0.68 | -0.95±0.83 | 0.60 | -1.3±1.22 | -1.81±1.34 | 0.90 |
| Waist circumference | -3.14±1.34 | -5.00±1.01 | 0.25 | -3.98±1.32 | -4.63±1.48 | 0.72 |
| Hip circumference | -2.20±1.49 | -3.88±1.17 | 0.35 | -5.8±1.15 | -3.38±1.61 | 0.18 |
| Waist/hip ratio | -1.14±1.05 | -5.11±3.62 | 0.38 | 0.46±0.98 | -1.25±0.79 | 0.18 |
| Fat mass | -0.76±0.31 | -0.96±0.23 | 0.62 | -0.89±0.25 | -1.46±0.40 | 0.20 |
| Total water | -0.0007±0.16 | +0.04±0.11 | 0.80 | -0.53±0.14 | -0.26±0.24 | 0.32 |
| Body cell mass | -0.11±0.10 | -0.18±0.07 | 0.60 | -0.63±0.16 | -0.30±0.21 | 0.19 |

Data are Mean±SEM.
